# Perinatal outcomes of growth -restricted fetus with abnormal umbilical artery doppler waveform; a global systematic review and meta-analysis

**DOI:** 10.1186/s12884-026-09534-y

**Published:** 2026-07-28

**Authors:** Anteneh Gashaw, Mulat Ayele, Yonas Abebe, Muluken Demeke, Belete Destaw, Addisu Getnet, Yayeh Adamu

**Affiliations:** 1 Department of Midwifery, College of Medicine & Health Sciences, Dilla University, Dilla, Ethiopia; 2 Department of midwifery, collage of health science, woldia university, Woldia, Ethiopia; 3 Department of Anesthesiology, College of Medicine & Health Sciences, Dilla University, Dilla, Ethiopia

**Keywords:** IUGR, Affected doppler finding, Doppler ultrasound, Perinatal outcome, Umbilical artery

## Abstract

**Introduction:**

Intrauterine growth restriction (IUGR) is associated with increased perinatal risks, including stillbirth, NICU admission, and neonatal complications. Doppler ultrasound helps diagnose abnormal fetal circulation in high-risk pregnancies, distinguishing IUGR from simple small-for-gestational-age fetuses. This systematic review and meta-analysis aim to assess the perinatal outcomes of IUGR with abnormal umbilical artery Doppler findings, providing valuable insights for improving risk management and healthcare practices.

**Method:**

A systematic review and meta-analysis were conducted in adherence to the PRISMA guidelines. Comprehensive literature searches were performed across several global databases, including PubMed, Google Scholar, ScienceDirect, Web of Science, Scopus, and African Online Journal databases. The pooled estimates were calculated using a weighted inverse variance random-effects model. To assess heterogeneity among the included studies, the Cochrane Q-test and I² statistics were utilized. Publication bias was examined using a funnel plot and Egger’s test.

All statistical analyses were performed using Stata version 17 software.

**Result:**

A total of 1,003 articles were initially identified, from which 36 studies met the inclusion criteria and were included in the final meta-analysis. Overall, data from 8,624 women were analyzed. The pooled perinatal mortality rate among fetuses with intrauterine growth restriction (IUGR) and abnormal umbilical artery Doppler findings was 16.54% (95% CI: 12.93–20.15). The pooled NICU admission rate among these neonates was 69.37% (95% CI: 58.52–80.21), indicating a substantial burden on neonatal care services. Furthermore, the pooled prevalence of reversed end-diastolic flow (REDF) in the umbilical artery among IUGR fetuses was 22.03% (95% CI: 13.44–30.62). Subgroup analysis revealed that the highest pooled estimates of perinatal mortality were observed in Europe and Asia, whereas the lowest rates were reported in Australia. In terms of REDF prevalence, the highest estimates were found in studies conducted in North America.

**Conclusion:**

This meta-analysis identified perinatal death, NICU admission, and low APGAR score as the most common adverse outcomes associated with IUGR and abnormal Doppler findings. The pooled perinatal mortality rate was 16.54%, which is notably high compared to other causes of perinatal mortality.

**Supplementary Information:**

The online version contains supplementary material available at https://doi.org/10.1186/s12884-026-09534-y.

## Introduction

When a fetus’ weight falls below the 10th percentile of the accepted threshold for weight to gestational age, this condition is referred to as “intrauterine growth restriction” (IUGR) [1]. The fetus is then classed as either constitutionally small or small for gestational age; The latter category may be the more serious one and could lead to early death [1, 2]. Its primary cause is a pathologic slowing of foetal growth, which prevents the foetus from growing to its full potential [3].

Doppler ultrasound of the fetal vasculature must be performed during high-risk pregnancies, such as those with suspected small for gestational age, preeclampsia eclampsia syndrome, fetal anemia and umbilical cord abnormality, to detect an aberrant circulation in the fetus [4], It is also used to distinguish between fetuses with tiny for date and the existence of growth retardation [2, 4], as well as to identify the course of disease in the uterus, and it is one way to decide how to handle a specific instance [2].

Pregnancies complicated by IUGR commonly require hospitalization because these IUGR fetuses are at elevated risk for adverse perinatal and neonatal outcomes as well as issues in early infancy [2, 5]. Perinatal morbidity and mortality have increased in the fetus with an abnormal umbilical Doppler waveform this might be decreased by using a vascular Doppler waveform as a diagnosis method [4, 6].

About 30 million babies worldwide experience IUGR each year, which is present in 23.8% of newborns. In developing regions, 75% of all exposed infants were born [7, 8] and the incidence of IUGR is between in the range of 3% − 10% for all pregnancy and this figure vary with different group of mother with a rate of 3% -5% for healthy mother and 25% and greater for mother with high risk like hypertensive disorder [3, 9].

Numerous studies have shown that still birth, NICU admission, low 5th minute Apgar score, neonatal hyperbilirubinemia [2], respiratory distress syndrome, mucoid aspiration syndrome, and neonatal sepsis are more frequently associated with IUGR whether the umbilical artery is affected or unaffected Waveform of Doppler [2, 4, 10, 11].

The main objective of this SRMA is to comprehensively analyze existing research studies to determine the potential perinatal outcomes in mothers diagnosed with intrauterine growth restriction (IUGR) accompanied by abnormal umbilical artery Doppler waveform. The significance of this research lies in its ability to provide valuable guidance to healthcare professionals in managing pregnancies with these conditions, aiding in risk stratification, informing evidence-based practice, and potentially influencing healthcare policy and resource allocation to improve outcomes for both mothers and babies.

## Method

### Study design and setting

A comprehensive evaluation of perinatal outcomes in cases of growth-restricted fetuses with abnormal umbilical artery Doppler waveforms was conducted globally through a SRMA. The study was adhere to the Preferred Reporting Items for Systematic Reviews and Meta-Analyses (PRISMA) guidelines, which aim to enhance transparency and accuracy in medical research reviews by providing checklists for conducting and reporting systematic reviews and meta-analyses across various fields [12].

Registration this SRMA (Systematic review and Meta-analysis) protocol was registered in PROSPERO database with registration id of CRD42023482486.

### Search strategies and sources of information

Before proceeding, we conducted a review of the PROSPERO database (http://www.library.ucsf.edu/) to check for any similar systematic review and meta-analysis (SRMA) studies. After thorough confirmation, no related studies are currently planned or completed in this database. Consequently, we submitted our protocol for registration in the PROSPERO database, was registered. A thorough search was conducted across various international databases, including PubMed, Google Scholar, PsycINFO, ScienceDirect, Web of Science, and the African Online Journal, to gather relevant information on this topic.

Additionally, the reference lists of published articles were reviewed to identify further pertinent publications that might not have been included in these databases. Gray literature was sourced from Google. We used the following searching strategy which includes MeSH term and key words for international database: “Perinatal outcome” OR “Neonatal Outcome” AND “IUGR” OR “Small for gestational age” AND “Affected Doppler” OR “abnormal umbilical artery Doppler waveform”.

### Eligibility criteria

#### Inclusion criteria

To be included in this SRMA, studies must focus on perinatal outcomes in cases of growth-restricted fetuses with abnormal umbilical artery Doppler waveforms and must be presented in English. There were no race-based restrictions, and the inclusion period was extended up to the date of the last search conducted March 6, 2025.

##### Study setting

Studies conducted across different regions of the world was considered.

##### Study design

Cohort, case-control, and cross-sectional quantitative studies with original data reporting at least one perinatal outcome of IUGR with affected Doppler was included.

##### Language

In this review, we were included articles published in English or those that have an English translation.

#### Exclusion criteria

Article with qualitative design and those quantitative study without reporting outcome variable was excluded.

### Measurements of outcome

The primary outcome of this systematic review and meta-analysis was to assess the perinatal outcomes associated with intrauterine growth restriction (IUGR) in fetuses with abnormal umbilical artery Doppler findings. The key perinatal outcomes considered included perinatal mortality, NICU admission, cesarean section rate, and low Apgar scores at birth and at five minutes. These outcomes were extracted from the included studies and pooled to estimate the overall impact of IUGR with abnormal Doppler findings on perinatal health. A secondary outcome of this review was to determine the global pooled prevalence of reversed end-diastolic flow (REDF) in the umbilical artery among fetuses diagnosed with IUGR.

#### Operational definition

Fetal growth restriction (FGR): A pathological condition in which a fetus fails to achieve its genetically determined growth potential, usually due to maternal, placental, or fetal factors. It implies an underlying abnormality restricting normal growth [7]. Small for Gestational Age (SGA): A descriptive, statistical term referring to a fetus or newborn whose estimated fetal weight or birth weight is below the 10th percentile for a given gestational age, regardless of whether the infant is healthy or growth-restricted [7].

Perinatal mortality was defined as the occurrence of stillbirth (fetal death at ≥ 28 weeks of gestation) or early neonatal death within the first seven days of life.

Neonatal intensive care unit (NICU) admission was defined as admission of the newborn to a specialized neonatal care unit immediately after birth or during the early neonatal period.

A low Apgar score was defined as an Apgar score of < 7 at 5 min after birth.

### Method of extraction of data

Initially, all studies obtained from the search were imported into EndNote Version X20 to manage duplicates. The articles were then exported to a Microsoft Excel spreadsheet. Using a standardized data extraction methodology developed in Excel, each author independently extracted all necessary information following the Joanna Briggs Institute (JBI) data extraction format. A detailed data extraction format was created, consisting of multiple columns, each aligned with the components of the JBI format. To resolve any discrepancies among authors during data extraction, the authors collectively re-extracted data from the main articles after a discussion.

### Quality assessment

To assess the quality of each study incorporated in this SRMA, we used Grade analysis evaluation of evidence.

### Data processing and analysis

Data extracted from each study using Microsoft Excel were transferred to STATA version 18 for formal analysis. Given the anticipated heterogeneity, a random-effects model was employed. The results were presented through graphs, charts, tables, texts, and forest plots. Egger’s statistical test and funnel plots were utilized to assess the presence of publication bias, with a P-value less than 0.05 indicating significant bias. Heterogeneity was measured using I² tests and categorized as low, moderate, or high if the values were 70%, respectively [13].

### Result

### Study selection

**Fig. 1:**
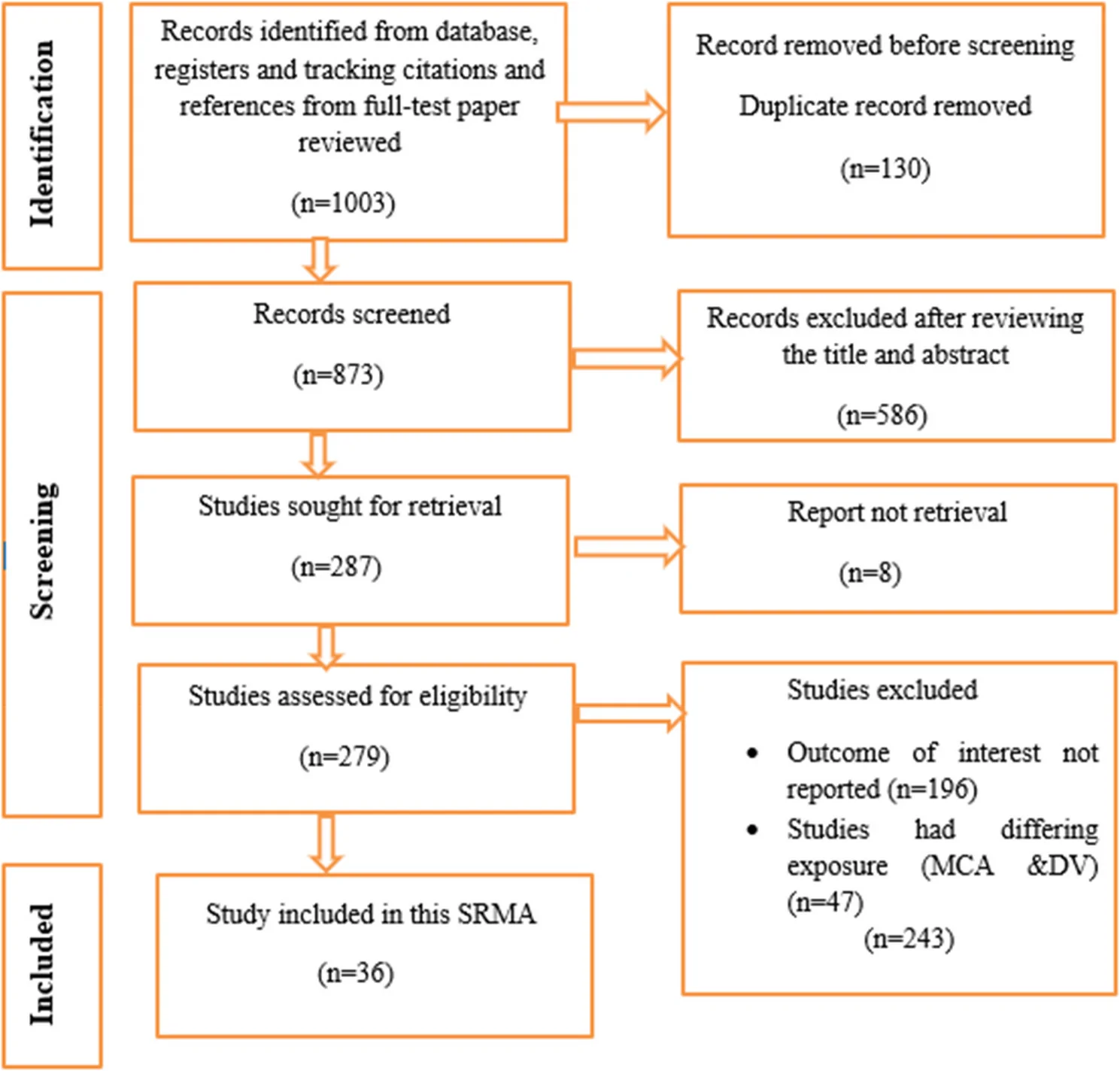
Study selection process in the study of perinatal outcome among IUGR fetus with Affected doppler finding; a systematic review and meta-analysis of global literature

Our search strategy encompassed African Journals Online, Google Scholar, PubMed, ScienceDirect, Web of Science, and Scopus, yielding a total of 1,003 articles—980 from databases and 23 through citation tracking and reference checks from full-text articles. After removing duplicates, 873 articles remained. Screening of titles and abstracts led to the exclusion of 484 and 102 articles, respectively. The remaining 287 full-text articles were assessed in detail based on the inclusion criteria. Of these, 251 were excluded for various reasons: 8 due to lack of access to the full text, 47 because they focused on a different exposure (MCA & DV) or were qualitative in nature, and 196 for not reporting the relevant outcome variables. In the end, 36 articles fulfilled the eligibility requirements and were included in the final systematic review and meta-analysis (Fig. 1).

### Characteristics of included articles

This systematic review and meta-analysis included 36 studies [2, 11, 14–47]. Among these, 20 studies (55.56%) were conducted in Asia, six in Europe, five in North America, four in Africa, and one in Australia. Regarding study design, 23 studies utilized a cohort design, six were nested case-control studies, another six were cross-sectional studies, and one study followed a case-control design.

A total of 8,624 women with intrauterine growth restriction (IUGR) participated in the study, with sample sizes ranging from 18 to 1,893. The included studies were published between 1998 and 2023 in the Gregorian calendar.

Concerning umbilical artery Doppler findings, the most severe abnormality observed was reversed end-diastolic flow (REDF), with its prevalence ranging from 2% to 46% across the studies. One of the most commonly reported perinatal outcomes was perinatal mortality, which ranged from 1% to 46%. The lowest perinatal mortality rate was reported in the United States, while the highest was recorded in Germany (Table 1).

**Table 1:** Characteristics of included studies in the systematic review and meta-analysis on prenatal outcomes of IUGR with Affected Umbilical artery doppler finding with a global literature

s.no	Author	Year	Region	study design	Sample size	With affected doppler	Reversed EDF
1	Abdelhalim et al. [14]	2014	Europe	Cohort	69	62	7
2	Adedo et al. [15]	2022	Africa	Cohort	42	42	4
3	Afroze et al. [16]	2015	Asia	Cohort	18	18	2
4	Afroze et al. [17]	2020	North America	Cohort	1893	143	2
5	Agarwal et al. [18]	2017	North America	Cohort	1893	22	13
6	Ali et al. [11]	2014	Asia	cohort	143	143	12
7	Bano et al. [19]	2010	Asia	cohort	50	20	
8	Bansal et al. [20]	2016	Asia	cohort	70	35	3
9	Baschat et al. [21]	2000	Europe	cohort	1085	139	
10	Baschat et al. [22]	2003	Asia	Cross-sectional comparative	66	33	
11	Channa et al. [23]	2023	North America	cohort	19	12	
12	Gaikwad et al. [24]	2018	Europe	Cross-sectional	255	112	6
13	Gerber et al. [25]	2006	Asia	Nested case-control	120	60	
14	Helmy et al. [26]	2021	Europe	Cross-sectional	75	75	
15	Hidaka et al. [27]	2017	Australia	cohort	119	70	12
16	Lewkowitz et al. [28]	2019	Asia	Nested case-control	60	30	
17	Lewkowitz et al. [29]	2021	Asia	cohort	60	28	
18	Magudapathi et al. [30]	2022	North America	cohort	61	24	
19	Mahale et al. [31]	2015	Asia	cohort	100	45	
20	Malhotra et al. [32]	2006	Asia	Nested case-control	200	59	
21	Morsing et al. [33]	2021	Africa	cohort	170	37	
22	Noor et al. [34]	2016	Asia	cohort	100	30	
23	Ozcan et al. [35]	1998	Europe	cohort	310	163	6
24	Ozyüncü et al. [36]	2010	Asia	cohort	172	78	
25	SARWAR et al. [37]	2021	Europe	cohort	69	62	
26	Schwarze et al. [38]	2005	Africa	cohort	42	42	42
27	Shand et al. [39]	2009	Asia	cohort	18	18	
28	Shivaprasad et al. [40]	2017	North America	cohort	1893	143	
29	Singh et al. [41]	2016	North America	cohort	1893	22	
30	Sterne et al. [42]	2001	Asia	cohort	143	143	
31	Sumathi et al. [43]	2013	Asia	cohort	50	20	
32	Thomas et al. [44]	2018	Asia	cohort	70	35	2
33	Tolu et al. [2]	2020	Europe	cohort	1085	139	
34	Ullah et al. [45]	2023	Asia	Cross-sectional comparative	66	33	
35	Yildirim et al. [46]	2008	North America	cohort	19	12	46
36	Zaman et al. [8]	2016	Europe	Cross-sectional	255	112	

### Perinatal outcomes of IUGR fetus with affected Umbilical Artery Doppler Waveform in the Global literature

The primary perinatal outcomes commonly reported in the global literature concerning intrauterine growth restriction (IUGR) with abnormal umbilical artery Doppler findings include perinatal mortality, low APGAR scores (less than 7 at both the first and fifth minutes), neonatal intensive care unit (NICU) admission, and cesarean delivery. In this systematic review and meta-analysis (SRMA), approximately 30 studies [2, 11, 14–19, 21–27, 30–33, 35, 36, 38, 39, 41–47] reported on perinatal mortality. The pooled estimate for perinatal mortality was 16.54 (95% CI: 12.93–20.15). The Cochrane heterogeneity index revealed substantial variability across studies (I² = 82.6%, *P* < 0.001), surpassing the 70% threshold, indicating significant heterogeneity. In addition, the 95% prediction interval was estimated (12.93–20.15) to account for between-study heterogeneity and to reflect the expected range of true effects in a new study setting.

Given the substantial heterogeneity observed across the included studies (I² > 70%), a random-effects model was applied for all meta-analyses. Random-effect model is considered more appropriate than a fixed-effect model when true effect sizes are expected to vary across studies due to differences in study populations, settings, and methodologies.

**Fig. 2:**
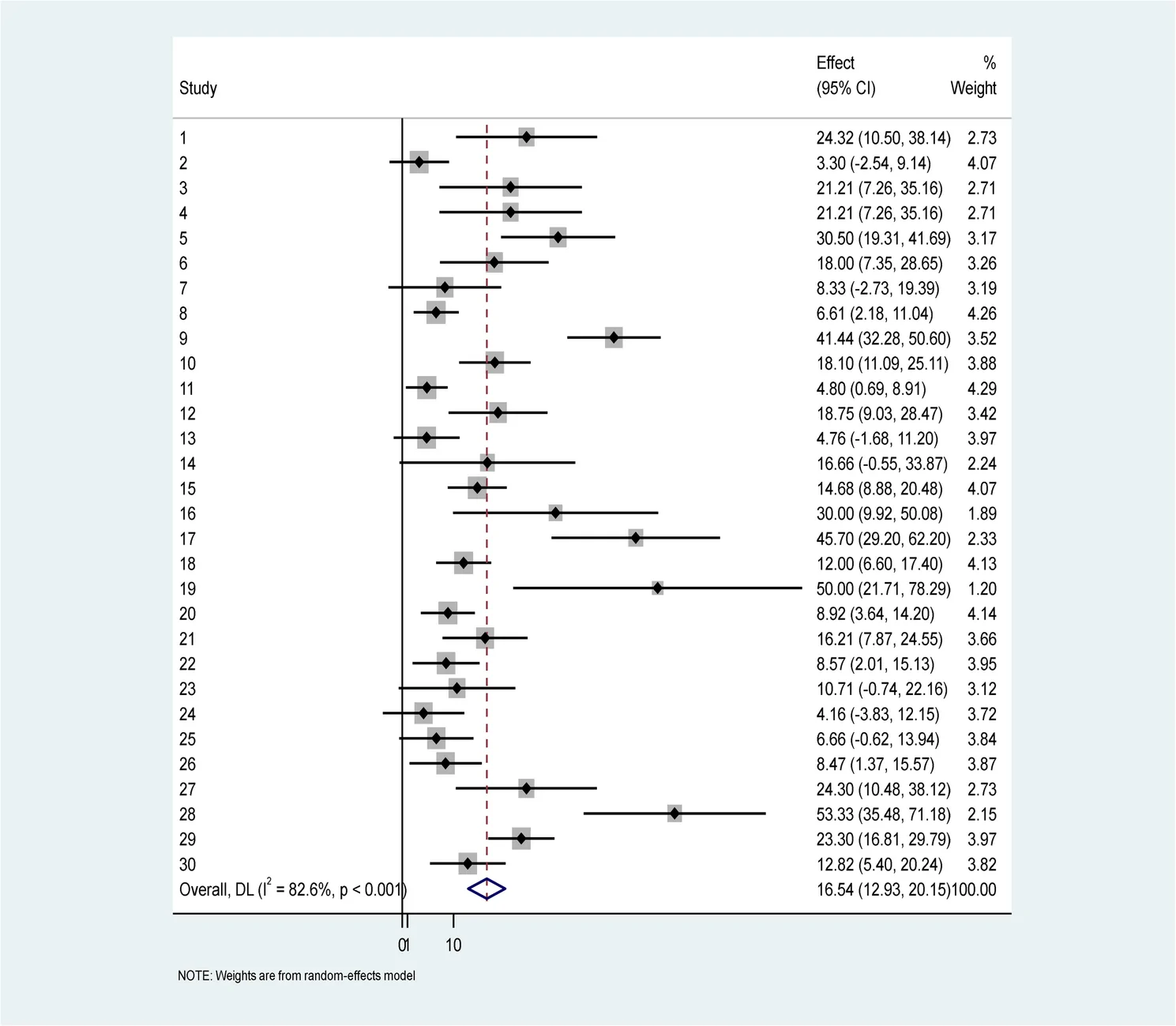
- The pooled estimate of perinatal mortality in study assessing perinatal outcomes among fetuses with intrauterine growth restriction (IUGR) and abnormal umbilical artery Doppler waveforms, a systematic review and meta-analysis based on global literature

**Fig. 3:**
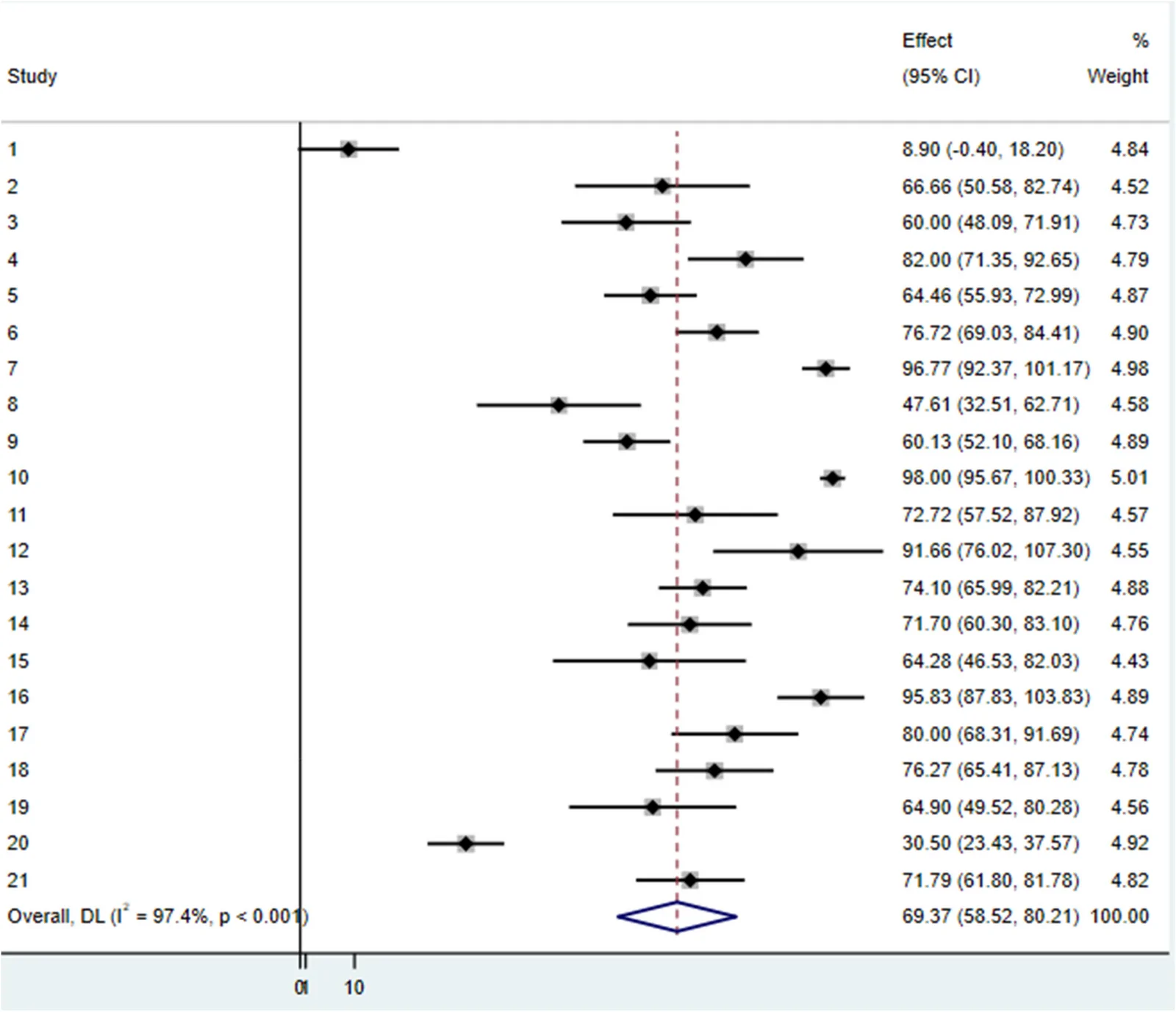
- The pooled estimate of NICU admission in study assessing perinatal outcomes among fetuses with intrauterine growth restriction (IUGR) and abnormal umbilical artery Doppler waveforms, a systematic review and meta-analysis based on global literature

As a result, a random-effects model was employed to account for this variation. Furthermore, subgroup analysis was conducted to investigate potential sources of heterogeneity and to offer a more detailed interpretation, with the findings illustrated in a forest plot (Fig. 2). Another perinatal outcome assessed in this meta-analysis was NICU admission. Among the 36 studies included in this systematic review and meta-analysis (SRMA), 21 studies [2, 11, 15, 16, 18, 21, 23, 25, 26, 30, 33, 35–37, 41–44, 46, 47] reported the rate of neonates admitted to the NICU. The pooled estimate of NICU admission among neonates with IUGR and abnormal Doppler findings was 69.37 (95% CI: 58.52–80.21). Additionally, significant heterogeneity was observed among the included studies, as indicated by the Cochrane heterogeneity index test (I² = 97.4%, *P* < 0.001) (Fig. 3).

### Umbilical Artery Doppler Finding

**Fig. 4:**
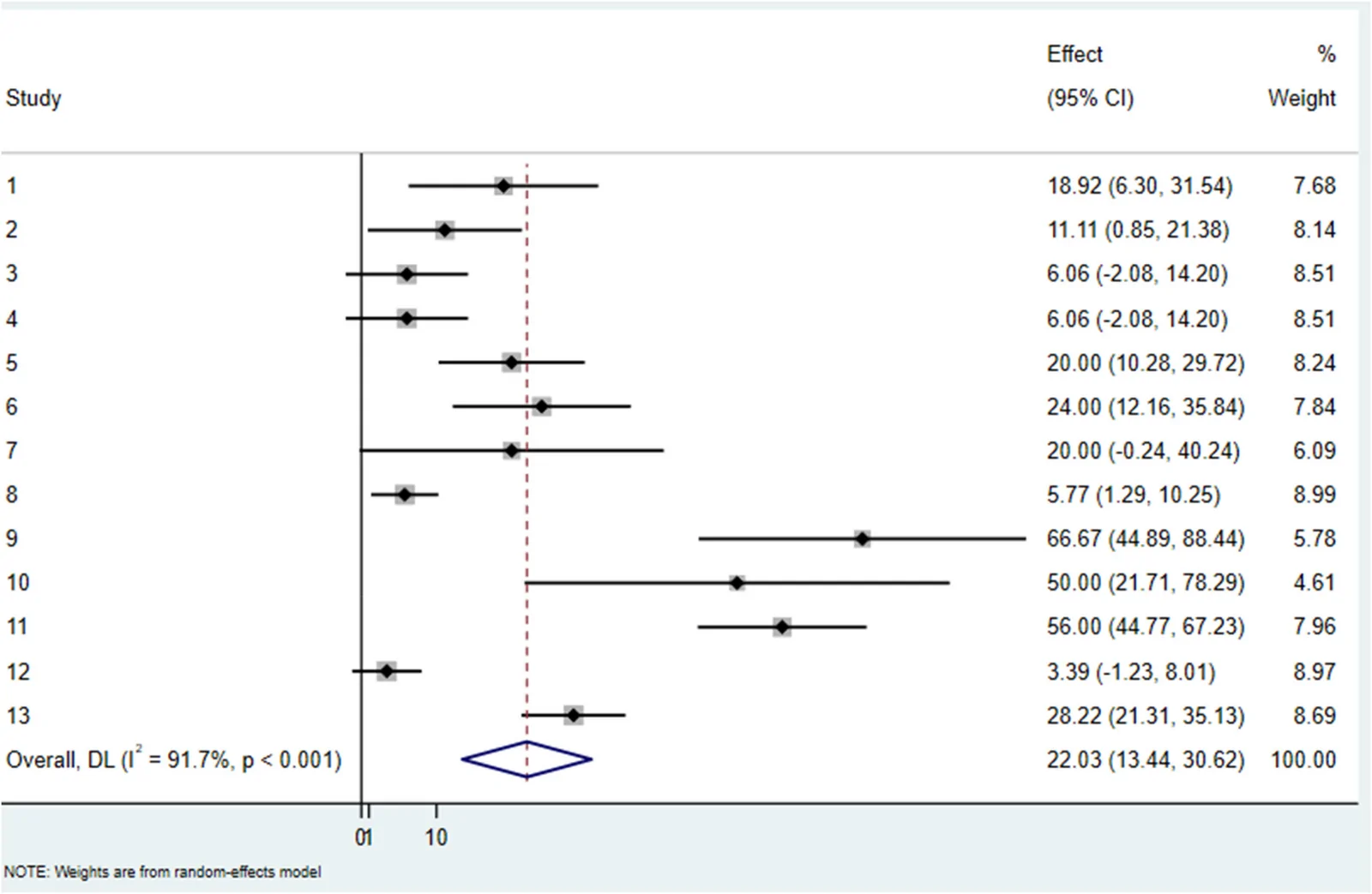
- The pooled estimate of REDF umbilical artery doppler finding in study assessing perinatal outcomes among fetuses with intrauterine growth restriction (IUGR) and abnormal umbilical artery Doppler waveforms, a systematic review and meta-analysis based on global literature

Among the various types of abnormal umbilical artery Doppler findings, the most serious is Reversed End-Diastolic Flow (REDF). Thirteen studies reported on the types of affected umbilical artery Doppler findings, and the pooled estimate indicates that approximately 22.03% (95% CI: 13.44–30.62) of fetuses were affected by REDF, according to global literature (Fig. 4). Moreover, the studies included in this analysis demonstrated substantial heterogeneity, as indicated by an I² value of 91.7% and a p-value of less than 0.001.

### Sub Group Analysis

**Fig. 5:**
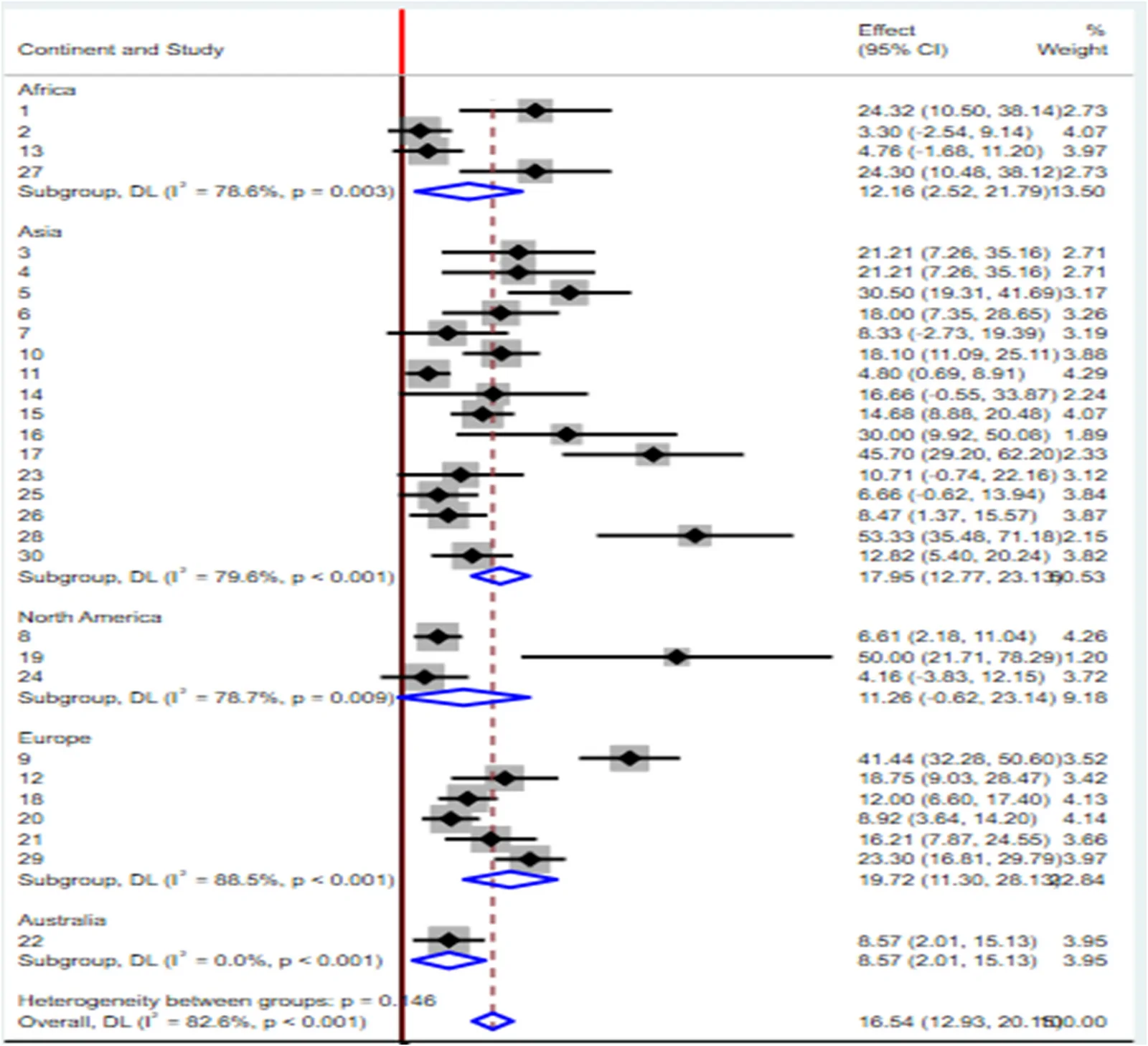
- Subgroup analysis by using continent on the pooled estimate of perinatal mortality among the study conducted on perinatal outcome among IUGR fetus with affected umbilical artery Doppler finding; a systematic review and meta-analysis with the global literature

**Fig. 6:**
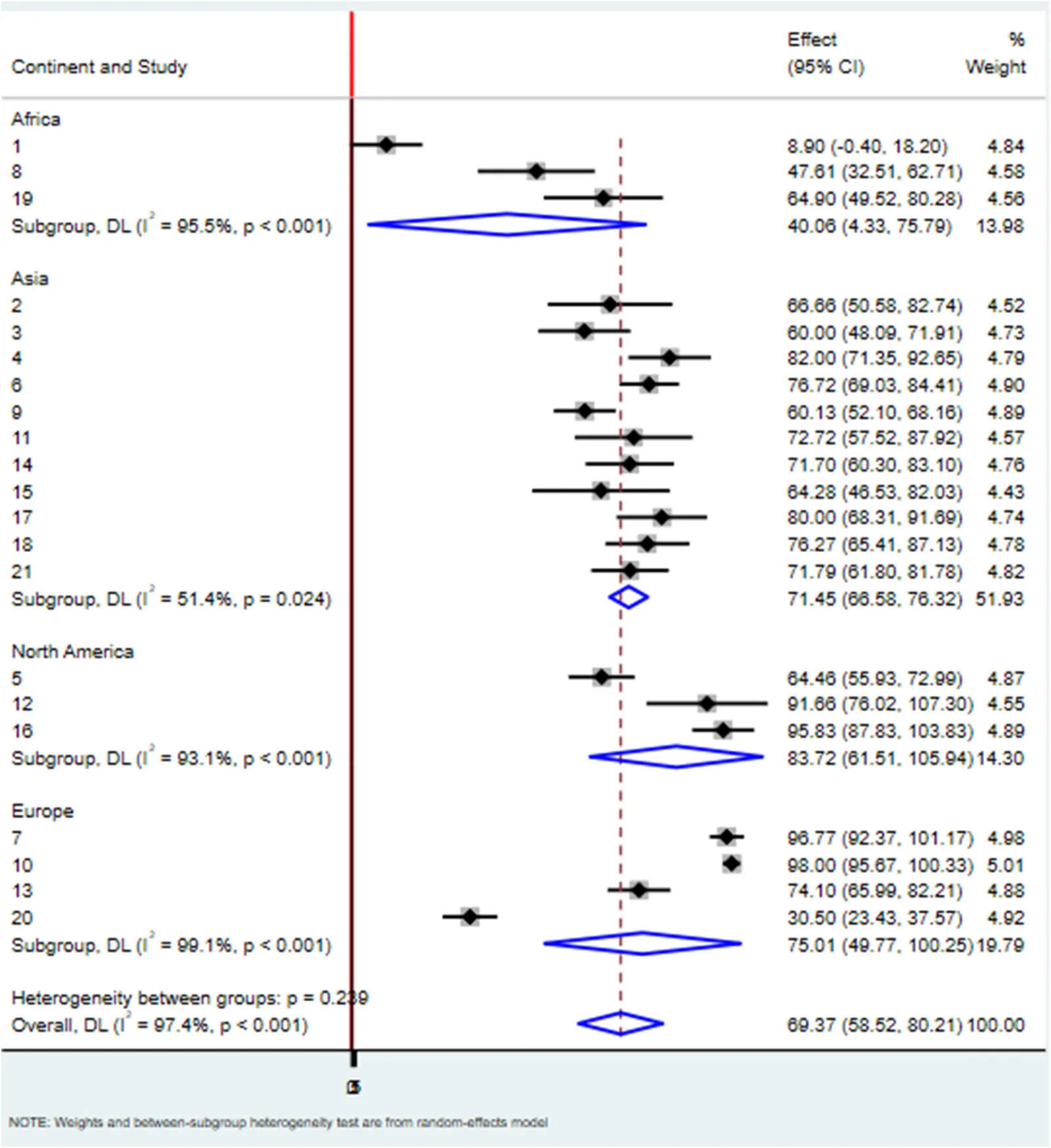
- Subgroup analysis by using continent on the pooled estimate of NICU admission among the study conducted on perinatal outcome among IUGR fetus with affected umbilical artery Doppler finding; a systematic review and meta-analysis with the global literature

**Fig. 7:**
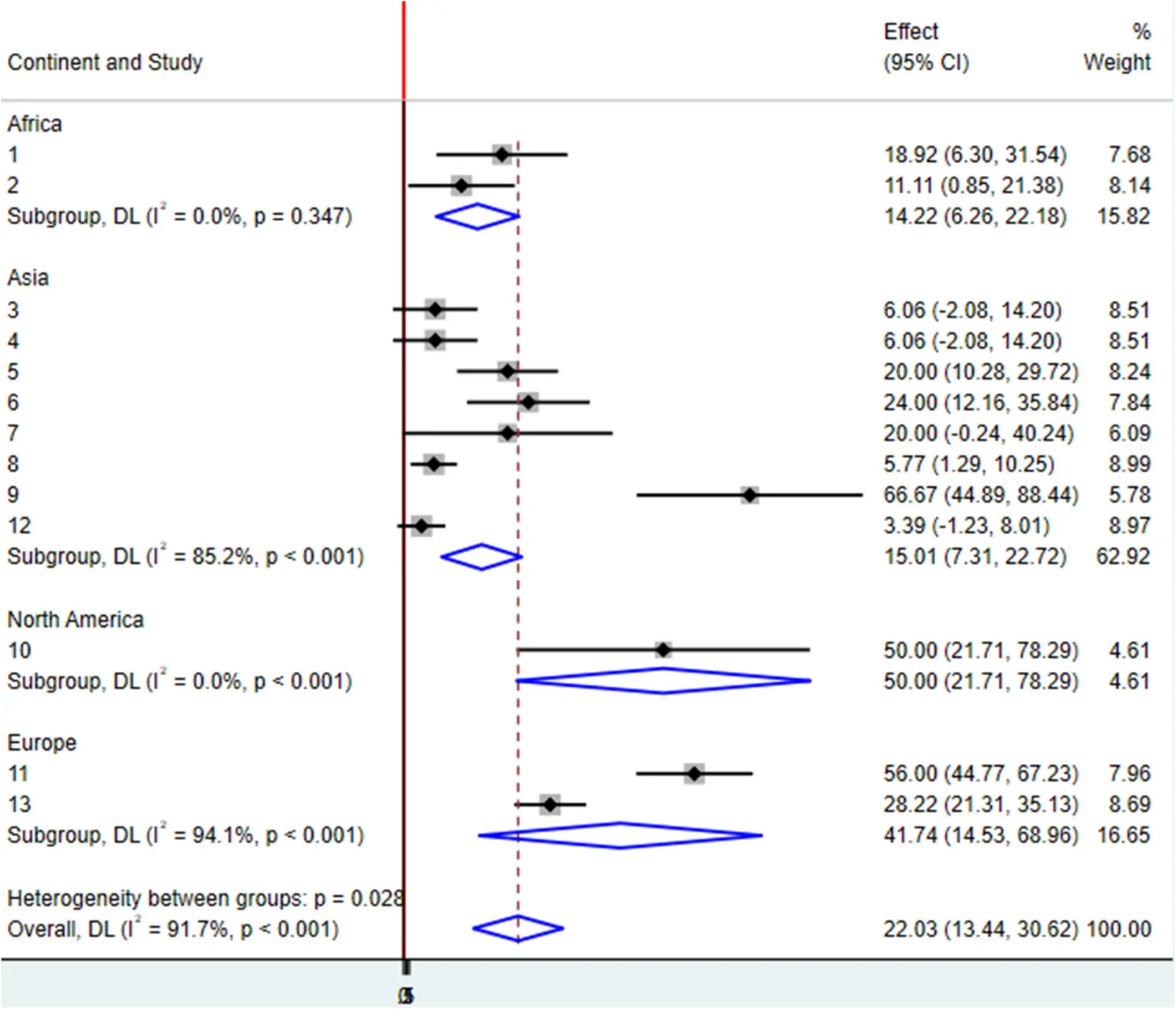
- Subgroup analysis by using continent on the pooled estimate of REDF umbilical artery doppler finding among the study conducted on perinatal outcome among IUGR fetus with affected umbilical artery Doppler finding; a systematic review and meta-analysis with the global literature

Due to the significant heterogeneity among the included studies, as indicated by (I² = 82.6%, *P* < 0.001), a subgroup analysis was conducted based on continent. The analysis revealed that the highest pooled estimates of perinatal mortality were observed in Europe and Asia, with rates of 19.76 (95% CI: 11.30–28.13) and 17.95 (95% CI: 12.77–23.13), respectively. In contrast, the lowest estimate was recorded in Australia, with a pooled perinatal mortality rate of 8.57 (95% CI: 2.01–15.13), Despite these regional differences, the test for subgroup differences (interaction test) was not statistically significant (*P* = 0.146), indicating that continent did not significantly account for the heterogeneity observed in perinatal mortality outcomes. (Fig. 5). Similar to those studies included in estimation of perinatal mortality a subgroup analysis was undergo for those studies included in the estimation of NICU admission therefore the highest pooled estimation rate was reported in North America which is 83.72 (95% CI: 61.51–105.94) were as the lowest rate was reported in Africa with 40.06 (95% CI: 4.33–75.79), Despite these regional differences, the test for subgroup differences (interaction test) was not statistically significant (*P* = 0.239), indicating that continent did not significantly account for the heterogeneity observed in perinatal NICU admission outcome (Fig. 6). A subgroup analysis of REDF umbilical artery Doppler findings based on continent revealed notable variation. The highest prevalence was reported in North America, with a rate of 50%, while the lowest was observed in Africa, at 14.22%, the test for subgroup differences (interaction test) was statistically significant (*P* = 0.028) This significant interaction suggests that continent may partly explain regional differences in REDF prevalence among IUGR fetuses with abnormal Doppler findings (Fig. 7).

### Publication Bias

In this systematic review and meta-analysis, statistical assessments, including funnel plot visualization and Egger’s regression test, were conducted to evaluate potential publication bias.

**Fig. 8:**
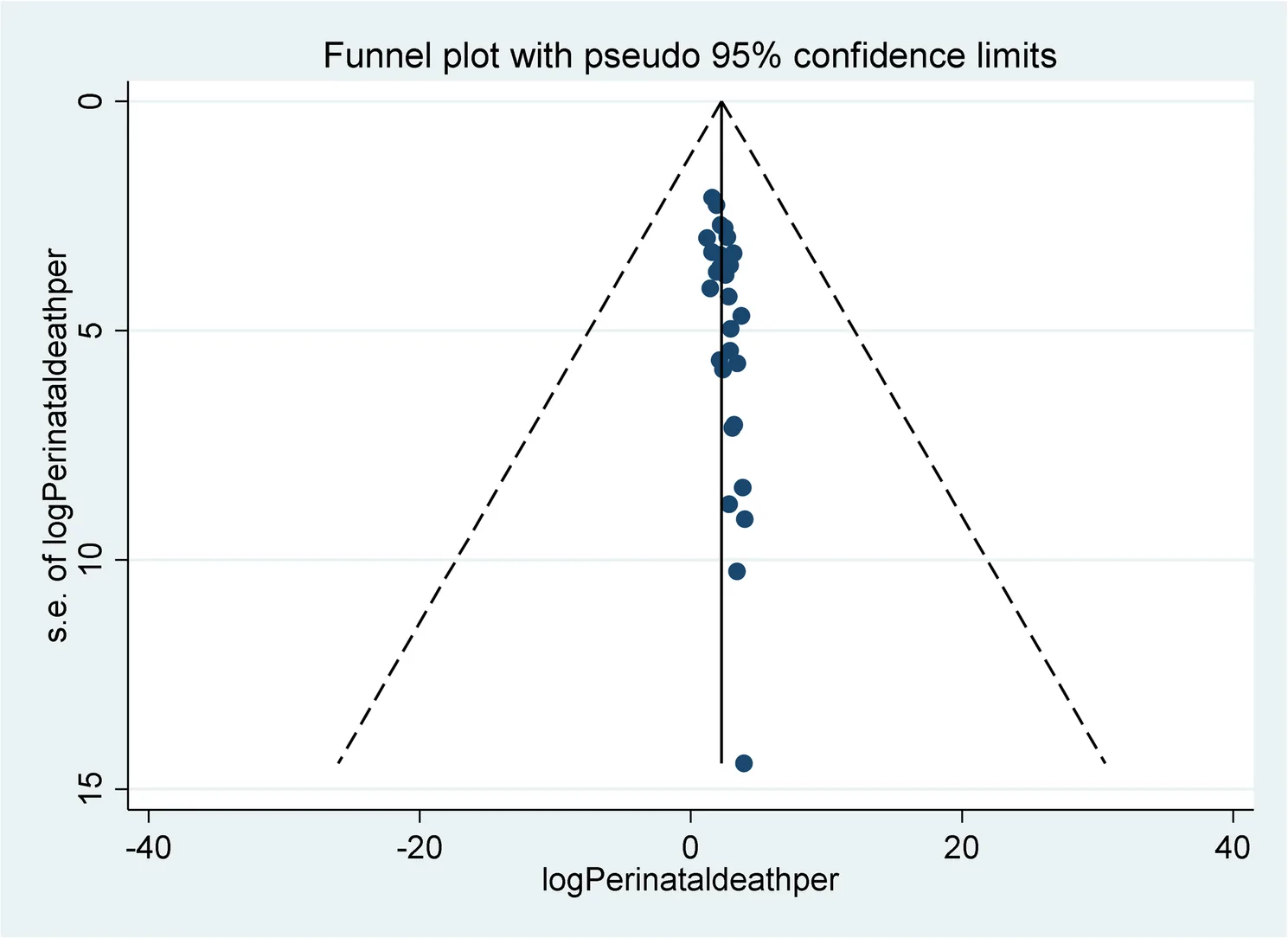
- Funnel plot show the distribution of studies for the estimation of perinatal mortality in study of perinatal outcome among IUGR fetus with affected Doppler finding: a systematic review and meta-analysis of a global literature

**Fig. 9:**
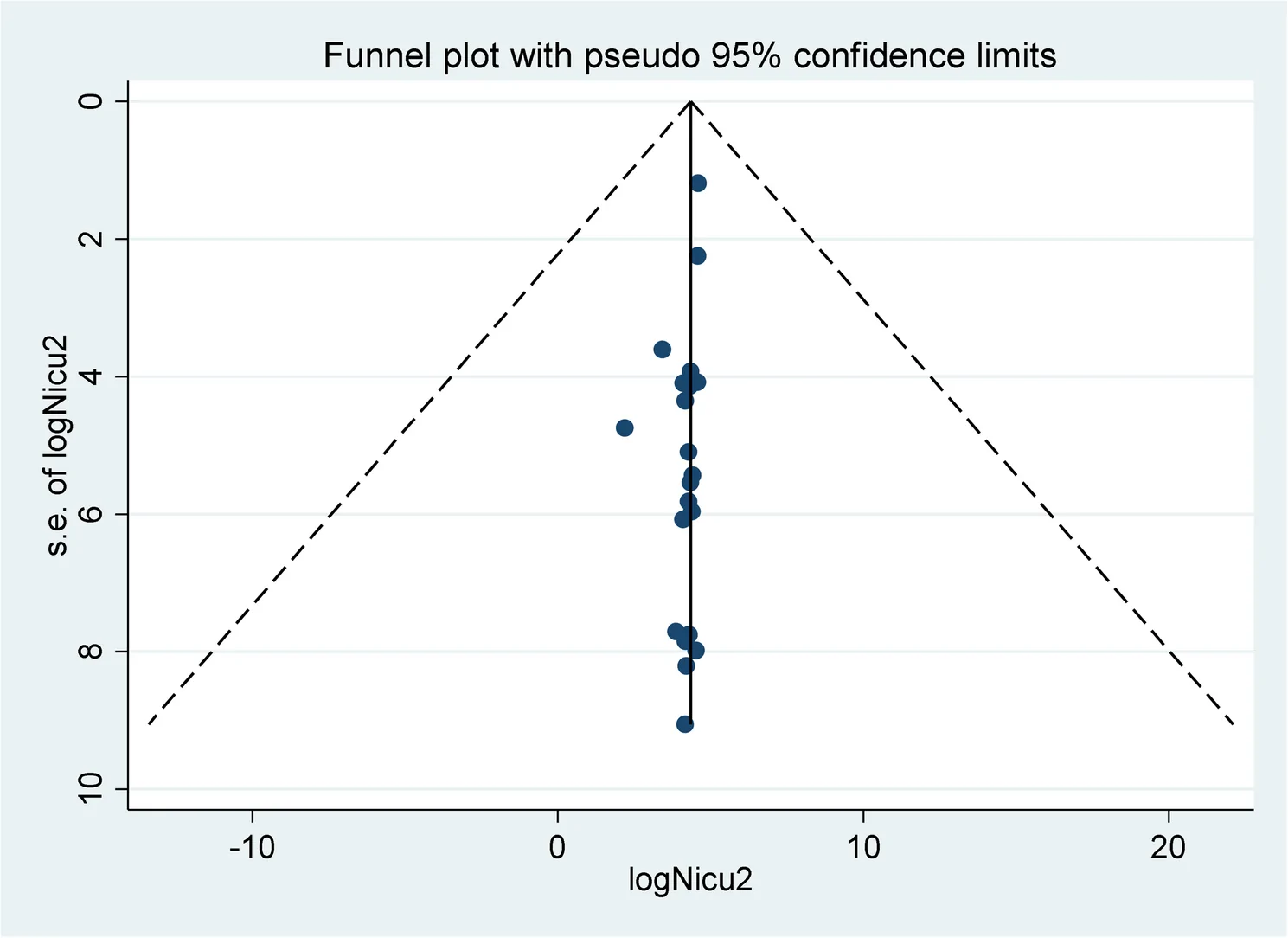
- Funnel plot show the distribution of studies for the estimation of NICU admission in study of perinatal outcome among IUGR fetus with affected Doppler finding: a systematic review and meta-analysis of a global literature

**Fig. 10:**
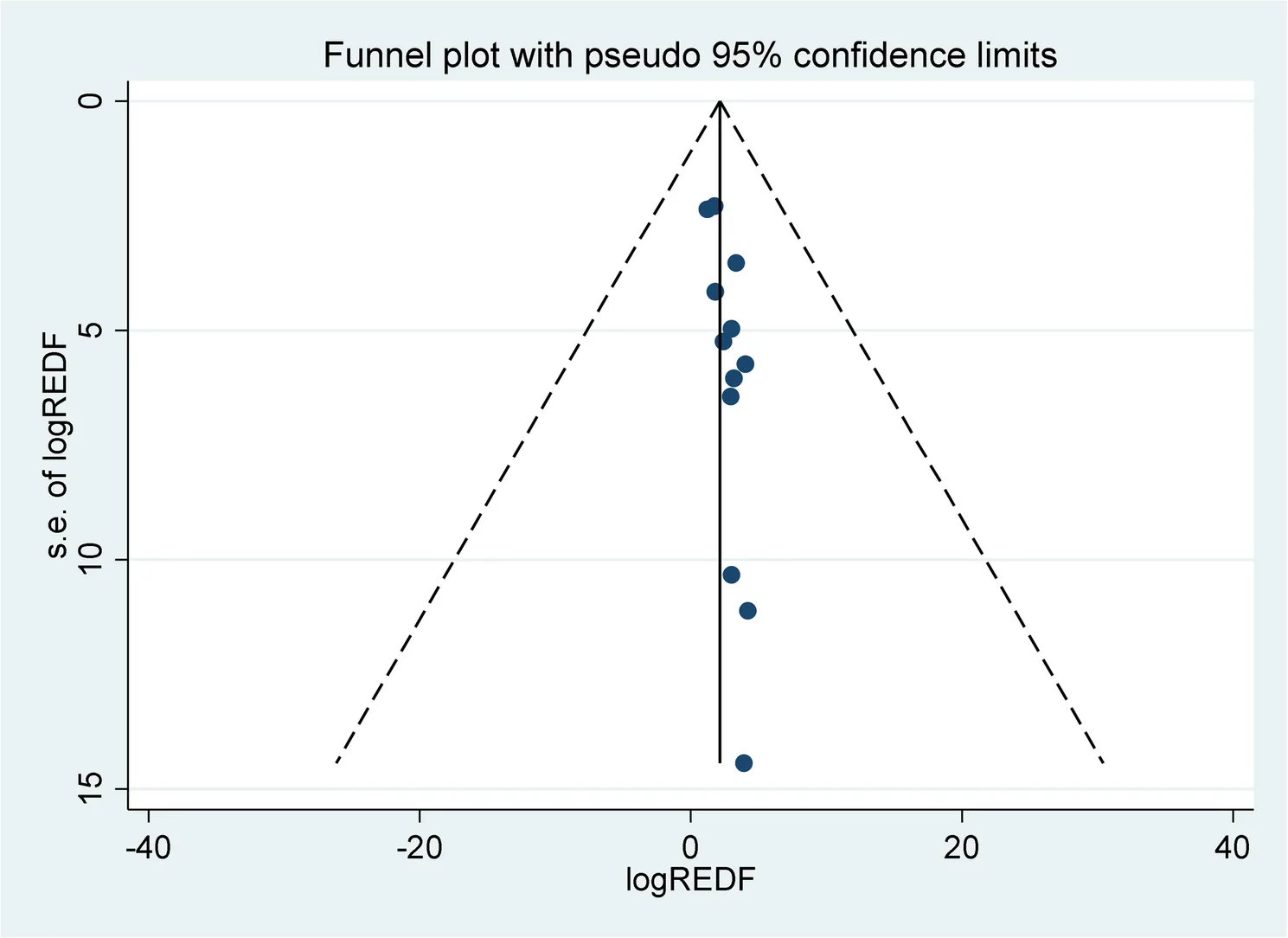
- Funnel plot show the distribution of studies for the estimation of REDF umbilical artery doppler finding in study of perinatal outcome among IUGR fetus with affected Doppler finding: a systematic review and meta-analysis of a global literature

For the studies estimating perinatal mortality in the global literature, the funnel plot appeared asymmetrical; however, Egger’s regression test was not statistically significant (*P* = 0.1, *p* > 0.05), indicating no strong evidence of publication bias (Fig. 8). Similarly, for the studies assessing NICU admission rates, the funnel plot appeared relatively symmetrical, and Egger’s test also showed no significant publication bias (*P* = 0.3) (Fig. 9). Furthermore, in the analysis of studies estimating the pooled prevalence of REDF, the funnel plot appeared asymmetrical, but Egger’s test remained insignificant (*P* = 0.1) (Fig. 10), again suggesting no statistical indication of publication bias. Visual inspection of funnel plots suggested asymmetry for some outcomes. However, Egger’s regression test did not indicate statistically significant small-study effects. Funnel plot asymmetry may reflect between-study heterogeneity, differences in study size, or methodological diversity rather than true publication bias. Therefore, the findings related to publication bias were interpreted with caution.

### Sensitivity Analysis

**Fig. 11:**
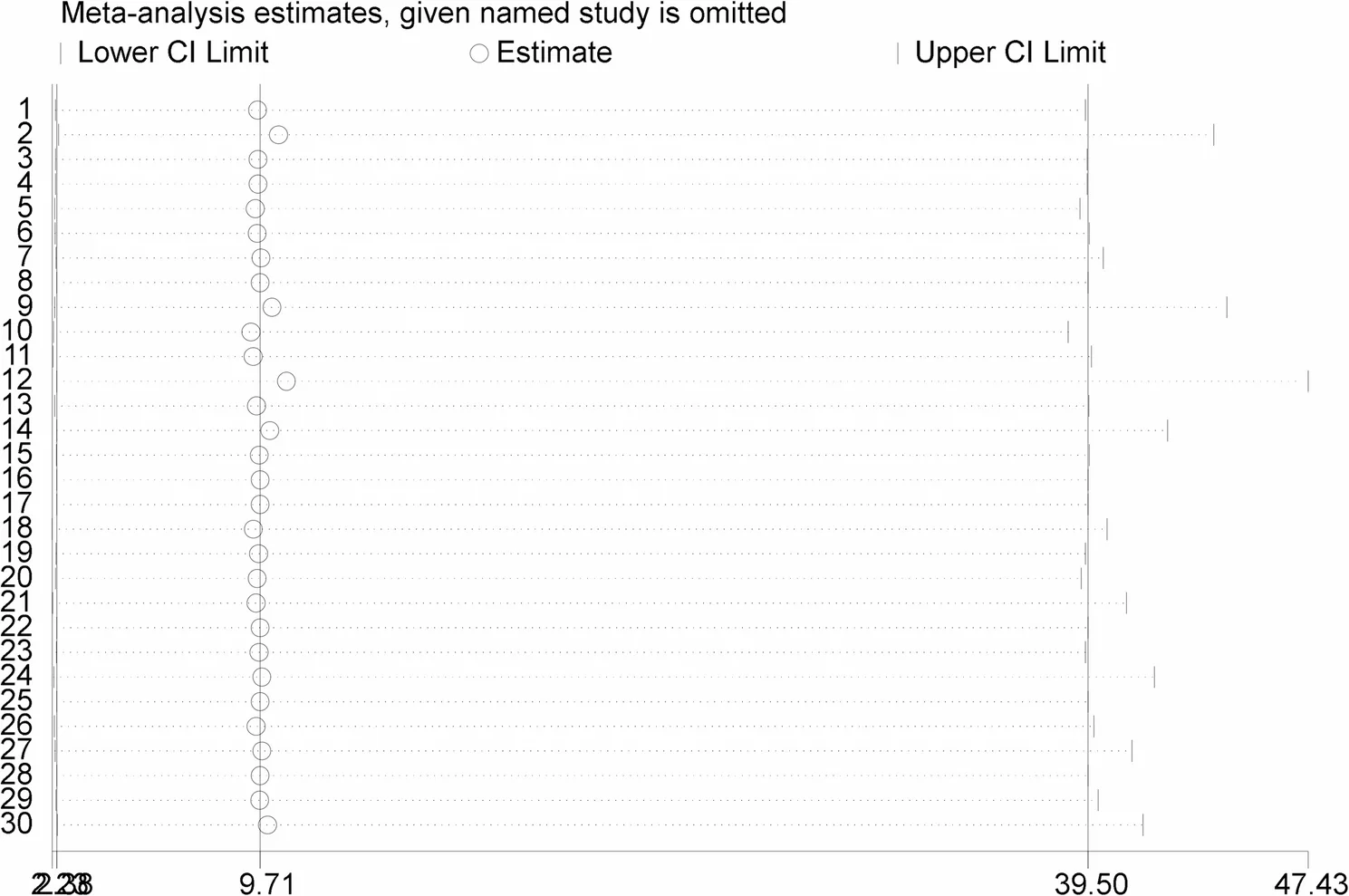
- The forest plot presents a sensitivity analysis of the pooled estimate of perinatal mortality in the study of perinatal outcome among IUGR fetus with affected umbilical doppler finding a systematic review and meta-analysis with a global literature

**Fig. 12:**
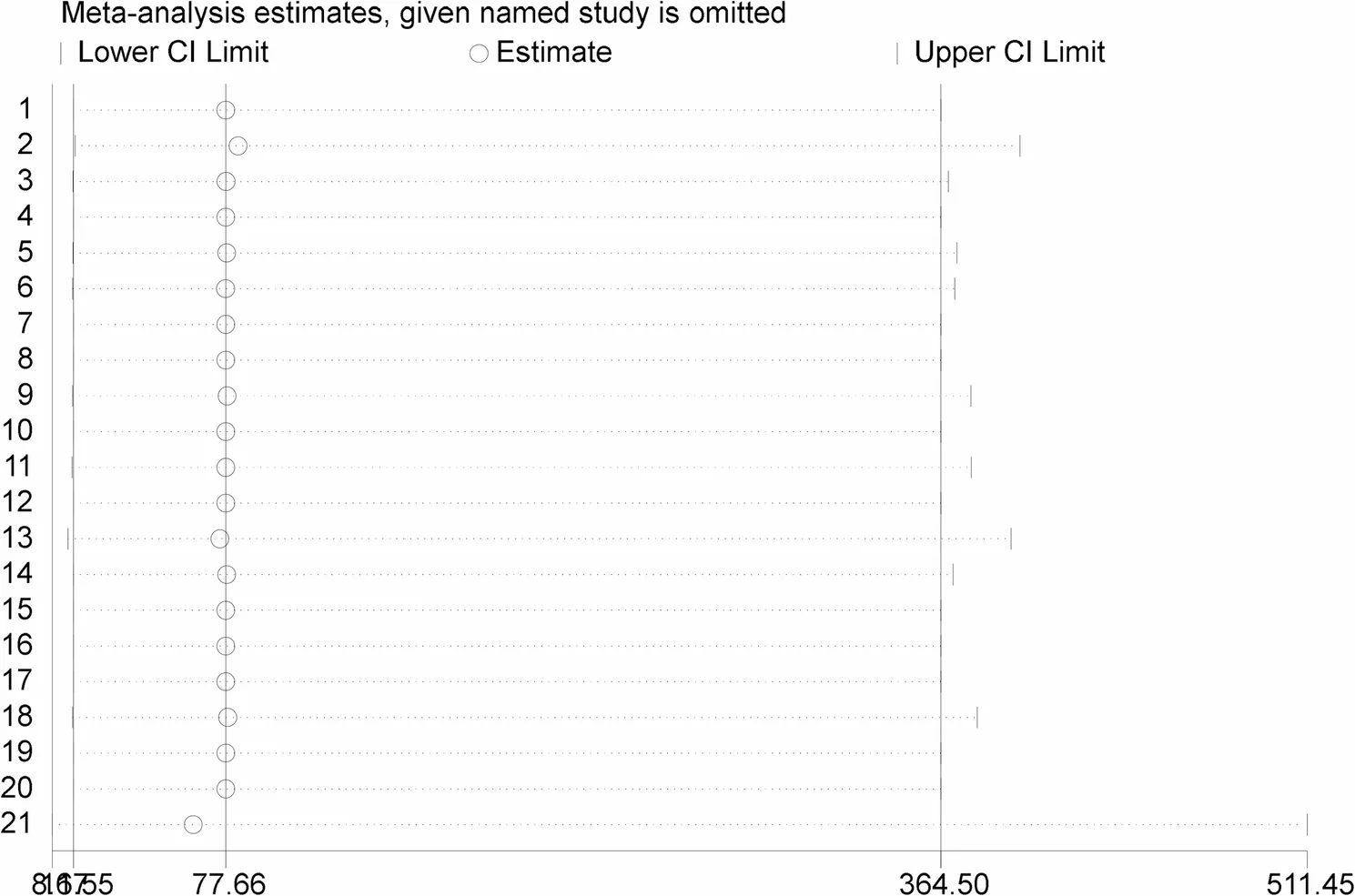
- The forest plot presents a sensitivity analysis of the pooled estimate of NICU admission in the study of perinatal outcome among IUGR fetus with affected umbilical doppler finding a systematic review and meta-analysis with a global literature

**Fig. 13:**
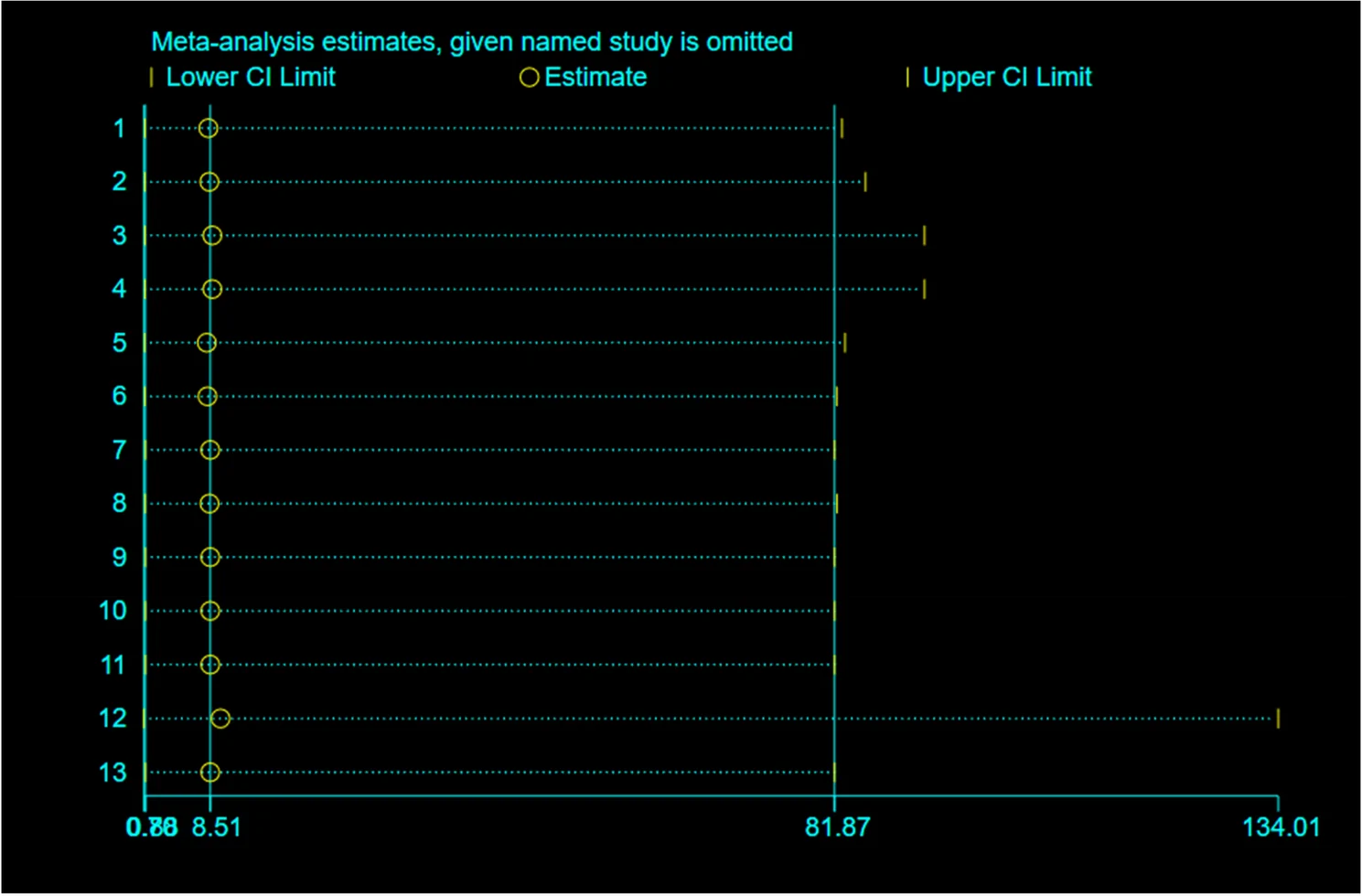
- The forest plot presents a sensitivity analysis of the pooled estimate of REDF umbilical artery doppler finding in the study of perinatal outcome among IUGR fetus with affected umbilical doppler finding a systematic review and meta-analysis with a global literature

A sensitivity analysis was performed to assess the influence of excluding individual studies on the pooled estimates of perinatal mortality, NICU admission, and REDF. The corresponding forest plots (Figs. 11, 12 and 13) illustrate how the overall proportions change when each study is sequentially removed. In these plots, each horizontal line represents the confidence interval resulting from the exclusion of a specific study, with the circles indicating the respective point estimates. The vertical line represents the overall pooled estimate. The results show that removing any single study does not lead to a substantial shift in the overall effect size, highlighting the consistency and robustness of the meta-analysis findings.

### Quality of the evidence

The GRADE approach was used to assess the certainty of evidence for each outcome. Evidence was downgraded for serious or very serious concerns related to risk of bias (e.g., observational study design and residual confounding), inconsistency (substantial statistical heterogeneity with I² >50%), and imprecision (wide confidence intervals or small numbers of studies). Evidence was upgraded when a large magnitude of effect was observed and when estimates were precise with consistent findings across studies. Despite the substantial pooled estimates, the certainty of evidence for all outcomes was rated as very low according to the GRADE framework. This downgrading was primarily due to serious risk of bias associated with observational study designs, marked inconsistency demonstrated by considerable heterogeneity across studies (I² > 80% for all outcomes), and imprecision in effect estimates. These findings indicate that while abnormal Doppler findings in IUGR are associated with significant adverse perinatal outcomes, confidence in the magnitude of these estimates remains limited, and the results should be interpreted with caution. Further well-designed prospective studies are required to improve the quality and certainty of the available evidence (Table 2).

**Table 2:** Grade analysis summary report for a meta-analysis of perinatal outcome among FGR fetus with affected umbilical Doppler finding

Outcome	Total studies (*N* = 36)	Pooled estimate (95% CI)	Heterogeneity	Overall certainty of evidence (GRADE)	Reasons for downgrading/upgrading
Perinatal death	30	16.54 (95% CI: 12.93–20.15).	(I² = 82.6%, *P* < 0.001)	Very Low	Risk of bias, serious inconsistency, imprecision
NICU admission	21	69.37 (95% CI: 58.52–80.21)	(I² = 97.4%, *P* < 0.001)	Very Low	Risk of bias, serious inconsistency, imprecision
REDF	13	22.03% (95% CI: 13.44–30.62)	I² =91.7%, *P* < 0.001	Very Low	Risk of bias, serious inconsistency, imprecision

## Discussion

The aim of this systematic review and meta-analysis was to determine the pooled estimates of various perinatal outcomes in cases of intrauterine growth restriction (IUGR) with affected umbilical artery Doppler findings, based on global literature. Among the perinatal outcomes commonly reported, perinatal death, NICU admission, Apgar scores less than 7, and cesarean section rates were the most frequently documented. This study specifically focused on estimating the pooled rates for perinatal mortality and NICU admission.

This meta-analysis provides robust global evidence on adverse perinatal outcomes among fetuses with intrauterine growth restriction (IUGR) and abnormal umbilical artery Doppler findings, expanding upon and strengthening the conclusions of previous individual studies and regional reviews. Earlier studies have consistently reported increased risks of perinatal mortality, NICU admission, and severe neonatal morbidity among IUGR fetuses with abnormal Doppler velocimetry; however, most were limited by small sample sizes, single-center designs, or geographic restriction. In contrast, by synthesizing data from 36 studies encompassing 8,624 pregnancies across multiple regions, the present meta-analysis offers more precise and generalizable pooled estimates.

The findings of this meta-analysis revealed that the pooled perinatal mortality rate was 16.54.

This rate was notably higher than the perinatal mortality observed in a meta-analysis of preterm birth but lower than the perinatal mortality reported in a meta-analysis of hypertensive disorders of pregnancy [48, 49]. Interestingly, this current finding is lower when compared to the perinatal mortality rate observed in a systematic review and meta-analysis that did not differentiate the underlying causes, which was approximately 58.35 [50]. This might be due to hypertensive disorders of pregnancy can likely be attributed to the complications these conditions cause in the placenta, which in turn can lead to IUGR [51]. Conditions like preeclampsia or gestational hypertension often impair placental function, leading to restricted fetal growth and an increased risk of perinatal mortality. These complications can cause poor oxygen and nutrient delivery to the fetus, further elevating the risks of adverse outcomes such as stillbirth, preterm birth, and other neonatal complications [52].

These comparative findings emphasize the significant, yet variable, impact of IUGR with abnormal umbilical artery Doppler findings on perinatal outcomes. The variability observed across different conditions underscores the critical need for tailored monitoring and management strategies in pregnancies affected by IUGR with abnormal Doppler findings, in order to optimize perinatal outcomes.

One of the perinatal outcomes estimated in this meta-analysis was NICU admission. The pooled estimate for NICU admission was found to be 69.37%. This rate was higher when compared to NICU admission rates in cases without an identifiable cause, yet it aligns with findings from meta-analyses conducted in different regions, particularly those involving pregnancy-induced hypertension and low birth weight [53–55]. The higher NICU admission rate in IUGR with abnormal umbilical artery Doppler findings is consistent with the fact that these pregnancies often experience complications such as preterm birth, respiratory distress, or low birth weight, all of which increase the likelihood of requiring NICU care [56]. The findings are also in line with other studies that highlight the significant need for specialized neonatal care in pregnancies complicated by conditions like pregnancy-induced hypertension and IUGR, which contribute to poorer perinatal outcomes [49, 50, 57].

AEDF is commonly seen in pregnancies with intrauterine growth restriction (IUGR) and is associated with higher risks of perinatal mortality, preterm birth, and neonatal morbidity. REDF, a more severe condition, occurs when blood flow in the umbilical artery reverses during diastole.

This condition reflects extreme placental insufficiency, indicating critical fetal compromise and an increased likelihood of stillbirth, neonatal death, or significant neonatal complications [58, 59]. In the present meta-analysis, the rate of Reversed End-Diastolic Flow (REDF) in the umbilical artery was analyzed in relation to obstetric complications: intrauterine growth restriction (IUGR). The pooled rate of REDF among pregnancies complicated by IUGR was estimated at 22.03, reflecting a strong association between REDF and severe placental insufficiency in growth-restricted fetuses. In contrast, the pooled prevalence of REDF among pregnancies affected by preeclampsia was slightly lower in some studies [60, 61]. Despite the lower rate, REDF in preeclamptic pregnancies still indicates significant vascular compromise and a heightened risk of adverse perinatal outcomes [58]. Comparatively, the higher rate of REDF in IUGR may be attributed to more pronounced and prolonged placental dysfunction characteristic of fetal growth restriction. These findings underscore the value of umbilical artery Doppler studies in stratifying risk and guiding clinical management in both IUGR and preeclamptic pregnancies, with particular vigilance warranted in IUGR cases where REDF is more prevalent [58, 59].

The pooled analyses demonstrated substantial heterogeneity (I² >80–90%), reflecting important clinical and methodological variability across the included studies. Potential clinical sources of heterogeneity include differences in gestational age at diagnosis and delivery, severity and type of Doppler abnormalities (e.g., umbilical artery absent or reversed end-diastolic flow, middle cerebral artery changes), timing and indications for delivery, and regional variations in obstetric and neonatal care practices. In addition, variation in study design, population characteristics, and outcome definitions may have contributed to the observed heterogeneity. Although a random-effects model was applied to account for between-study variability, the high level of heterogeneity limits the precision and generalizability of the pooled estimates. Therefore, the pooled results should be interpreted cautiously, and emphasis should be placed on the direction and consistency of associations rather than the exact magnitude of effect.

To our knowledge, this is the first global systematic review and meta-analysis focusing on perinatal outcomes among pregnancies complicated by intrauterine growth restriction (IUGR) with abnormal Doppler findings. Due to the absence of prior meta-analyses specifically targeting this subject, direct comparison with similar comprehensive studies was not feasible. Therefore, we compared our pooled results with relevant primary studies and meta-analyses addressing related issues. Several limitations should be considered when interpreting the findings of this meta-analysis. First, substantial heterogeneity was observed across most pooled outcomes, indicating considerable clinical and methodological diversity among the included studies.

Differences in study design, timing and technique of umbilical artery Doppler assessment, and outcome reporting likely contributed to this variability. Although random-effects models, subgroup analyses, sensitivity analyses, were used to address and characterize heterogeneity, unexplained heterogeneity persisted. Consequently, the pooled estimates represent average effects and may not be fully generalizable to all clinical settings.

## Conclusion

This meta-analysis identified perinatal death, NICU admission, and low APGAR score as the most common adverse outcomes associated with IUGR and abnormal Doppler findings. The pooled perinatal mortality rate was 16.54%, which is notably high compared to other causes of perinatal mortality. Furthermore, approximately 69.37% of neonates with IUGR and affected Doppler findings required NICU admission, underscoring the significant burden of this condition on neonatal health systems.

## Supplementary Information

Supplementary Material 1.

Supplementary Material 2.

## Data Availability

The findings of this Systematic Review and Meta-Analysis (SRMA) were derived from the collected data and analyzed according to the specified methods and materials. All pertinent data are comprehensively presented within the paper.

## Abbreviations

AOR: Adjusted odd ratio
IUGR: Intrauterine Growth Restriction
REDF: Reversed End Diastolic Flow
PRISMA: Preferred Reporting Items for Systematic Reviews and Meta-Analyses
SRMA: Systematic review and meta-analysis
