# Supplementary material for: Perinatal outcomes of growth -restricted fetus with abnormal umbilical artery doppler waveform; a global systematic review and meta-analysis
Source: BMC Pregnancy Childbirth. 2026 Jul 28;26:1215. doi: 10.1186/s12884-026-09534-y (PMC13700500; doi:10.1186/s12884-026-09534-y)
Supplement: Supplementary file 2 [file 12884_2026_9534_MOESM2_ESM.docx]

Figure 8: - Funnel plot show the distribution of studies for the estimation of perinatal mortality in study of perinatal outcome among IUGR fetus with affected Doppler finding: a systematic review and meta-analysis of a global literature.

Figure 9: - Funnel plot show the distribution of studies for the estimation of NICU admission in study of perinatal outcome among IUGR fetus with affected Doppler finding: a systematic review and meta-analysis of a global literature.

Figure 10: - Funnel plot show the distribution of studies for the estimation of REDF umbilical artery doppler finding in study of perinatal outcome among IUGR fetus with affected Doppler finding: a systematic review and meta-analysis of a global literature.

Figure 11: - The forest plot presents a sensitivity analysis of the pooled estimate of perinatal mortality in the study of perinatal outcome among IUGR fetus with affected umbilical doppler finding a systematic review and meta-analysis with a global literature.

Figure 12: - The forest plot presents a sensitivity analysis of the pooled estimate of NICU admission in the study of perinatal outcome among IUGR fetus with affected umbilical doppler finding a systematic review and meta-analysis with a global literature.

Figure 13: - The forest plot presents a sensitivity analysis of the pooled estimate of REDF umbilical artery doppler finding in the study of perinatal outcome among IUGR fetus with affected umbilical doppler finding a systematic review and meta-analysis with a global literature.
